# Noncontact atomic force microscopy III

**DOI:** 10.3762/bjnano.7.86

**Published:** 2016-06-30

**Authors:** Mehmet Z Baykara, Udo D Schwarz

**Affiliations:** 1 Department of Mechanical Engineering and UNAM-Institute of Materials Science and Nanotechnology, Bilkent University, Ankara 06800, Turkey; 2 Departments of Mechanical Engineering & Materials Science and Chemical & Environmental Engineering and Center for Research on Interface Structures and Phenomena (CRISP), Yale University, P.O. Box 208284, New Haven, CT 06520-8284, USA

**Keywords:** atomic force microscopy, scanning force microscopy

Intense interest in nanoscale science and technology has been the main driving force behind a large number of outstanding discoveries in the last few decades. It may not be an overstatement to claim that the development of the various scanning probe methods in the 1980s was the main pre-requisite for the fields of nanoscience and nanotechnology to take off and ultimately evolve to their current states. While scanning tunneling microscopy (STM) relies on quantum mechanical tunneling of electrons to enable the atomic-resolution imaging of (semi-)conducting sample surfaces, it was the atomic force microscope (AFM) that eventually allowed for nanometer-scale imaging of sample surfaces with no limitations on electrical conductivity. As such, the method was widely adopted shortly after its introduction in 1986 and today it is not unusual to have multiple AFMs available at research universities and R&D departments of industrial companies.

Despite their widespread use, a major drawback of traditional AFM instruments is the fact that they rely on the establishment of light contact between a sharp probe and the sample surface of interest for topographical imaging. This results unavoidably in the formation of a finite contact area and the loss of atomic-scale resolution. The invention of noncontact atomic force microscopy (NC-AFM) in 1994 offered an elegant solution to this problem: Instead of touching the sample surface, the probe hovers a short distance above while the micro-machined cantilever that the probe is attached to is oscillated at its resonance frequency. The attractive interaction forces acting between the outermost atoms of the probe apex and the atoms on the surface then cause a downshift in oscillation frequency, which is employed as the feedback signal during lateral scanning. In this way, the probe apex remains atomically sharp and it becomes possible to attain atomic-scale resolution on a wide variety of sample surfaces.

The method of NC-AFM has evolved significantly since its introduction and it is now possible to employ the technique to visualize the internal structure of individual molecules, controllably manipulate single atoms on surfaces, and measure potential energy landscapes with unprecedented resolution. Moreover, NC-AFM is not only limited to operation under ultrahigh vacuum and it can now be utilized to study the detailed structure and even the dynamic activity of biological molecules. To keep up with the rapid progress in this exciting field, since 1998 the NC-AFM community meets every year at the annual “International Conference on Non-Contact Atomic Force Microscopy” series, which is typically characterized by lively discussions on the latest technical and scientific developments related to NC-AFM. In parallel with the series of conferences, the last few years have seen the publication of two installments in the Thematic Series titled “Noncontact Atomic Force Microscopy” in the *Beilstein Journal of Nanotechnology* [1–2]. This Thematic Series focusing on NC-AFM complements two other series titled “Advanced Atomic Force Microscopy Techniques” [3–4] and “Scanning Probe Microscopy and Related Methods” [5], making the *Beilstein Journal of Nanotechnology* a well-recognized outlet for scanning probe microscopy research.

The current and third installment in the “Noncontact Atomic Force Microscopy” Thematic Series again demonstrates the constant development in the field. In particular, latest instrumental advances are highlighted in the form of a new design for a large-area SPM used for electrostatic force measurements, improvement of dynamic cantilever response by the utilization of reflective coatings and photothermal conversion layers, and the use of length extension resonators for NC-AFM operation in air. In addition, the ever increasing importance of simulations for dynamic AFM experiments is underlined via two contributions focusing on three-dimensional viscoelastic modeling as well as “sub-atomic” contrast formation on the prototypical Si(111)-7×7 surface.

To conclude, we would like to sincerely thank the authors for submitting their exciting work to the latest installment in the Thematic Series, the reviewers for their careful analysis and appraisal of submitted work, and the great editorial team at the *Beilstein Journal of Nanotechnology*, which provides our community with a unique, Open Access platform for the dissemination of research results.

Mehmet Z. Baykara and Udo D. Schwarz

Ankara, New Haven, May 2016
